# Role of solvents in the electronic transport properties of single-molecule junctions

**DOI:** 10.3762/bjnano.7.99

**Published:** 2016-07-22

**Authors:** Katharina Luka-Guth, Sebastian Hambsch, Andreas Bloch, Philipp Ehrenreich, Bernd Michael Briechle, Filip Kilibarda, Torsten Sendler, Dmytro Sysoiev, Thomas Huhn, Artur Erbe, Elke Scheer

**Affiliations:** 1 Physics Department, University of Konstanz, D-78457 Konstanz, Germany; 2 Institute of Ion Beam Physics and Materials Research, Helmholtz-Zentrum Dresden-Rossendorf, D-01328 Dresden, Germany; 3 Chemistry Department, University of Konstanz, D-78457 Konstanz, Germany

**Keywords:** electrochemical environment, mechanically controllable break junction, molecular electronics, polar solvent, single-molecule junctions

## Abstract

We report on an experimental study of the charge transport through tunnel gaps formed by adjustable gold electrodes immersed into different solvents that are commonly used in the field of molecular electronics (ethanol, toluene, mesitylene, 1,2,4-trichlorobenzene, isopropanol, toluene/tetrahydrofuran mixtures) for the study of single-molecule contacts of functional molecules. We present measurements of the conductance as a function of gap width, conductance histograms as well as current–voltage characteristics of narrow gaps and discuss them in terms of the Simmons model, which is the standard model for describing transport via tunnel barriers, and the resonant single-level model, often applied to single-molecule junctions. One of our conclusions is that stable junctions may form from solvents as well and that both conductance–distance traces and current–voltage characteristics have to be studied to distinguish between contacts of solvent molecules and of molecules under study.

## Introduction

The electronic transport properties of single-molecule junctions are actively investigated with the aim to utilize such junctions as functional building blocks in electronic devices [1–8]. A very fruitful method for gathering statistical information on the transport behaviour of single-molecule junctions is the repeated formation and breakage of atomic contacts immersed in a solution containing the molecules under investigation in a suitable solvent. The dissolution of the molecules in a solvent enables their diffusion to the metal electrodes and thus the repeated formation of new and independent junctions when separating the electrodes, signalled by the formation of steps in the conductance-vs-distance curves. The conductance *G*_J_ of these molecular junctions is then ascribed to the metal/molecule/metal junction alone, neglecting the contributions *G*_s_ of the solvents. This assumption is justified if *G*_J_ is much larger than *G*_s_. Except for the seminal studies by Grüter et al. [9–10] the role of the solvent for the transport properties of single-molecule contacts has been discussed only very recently [11–22]. The most obvious impact may be the change of the work function, because the tunnelling does not take place through vacuum states but through an electrolyte that alters the work function of the electrode and thereby affects the level alignment [9–10,13,21]. In general, a molecular junction is not formed in all of the breaking curves, but the majority of curves show smooth or noisy distance dependence. These curves are interpreted as tunnelling through the solvent that has no influence on the apparent conductance values of the molecules under study.

However, often the current–voltage (*I*–*V*) characteristics of these electrolyte tunnel junctions have shapes that are similar to those found in single-molecule junctions and are therefore not easy to distinguish from each other [11]. In principle this would be possible by setting up a correlation between distance and *I*–*V* curve, but in practice this is a difficult task, since often the distance between the electrode pairs cannot be measured precisely or only on a relative basis, because after breaking the single atom metal contact the distance between the electrodes can change rapidly by several angströms due to the sudden release of stress.

Furthermore, a Helmholtz double layer builds on the surface of a metal immersed in an electrolyte or a polar solvent, forming a capacitor [23]. When changing the bias this layer may become instable and thus influence the *I*–*V*s. Its impact can be reduced by protecting large areas of the electrodes with an insulating material and thus reducing the capacitance. Typically the electrode pair used in single-molecule junctions consists of a surface and a fine tip, if one uses a scanning tunnelling microscope [24–25] for forming the contacts, or even two fine tips when applying the mechanically controllable break junction (MCBJ) technique [26–28]. Also, when using planar electrodes, e.g., in the electromigration technique [29], the very ends show features with corrugations of atomic size.

The typical voltage applied when recording conductance histograms is 100 mV, *I*–*V*s are often taken in voltage ranges of ±1 V, and typical tunnel gaps have sizes in the order of single nanometres. As a result the local electrical fields may be of the order of 10^9^ V/m, possibly causing cleavage of the solvent or the molecule. Potential artefacts include the formation of stable conduction states with conductance values below those of single atoms, resulting in steps in the breaking curves also when using the pure solvent. Additionally, the solvent may also decorate the metal electrodes or is even incorporated and thus influence the geometry of the junctions that form.

In this work we investigate the influence of solvents on the interpretation of transport measurements of single-molecule junctions. For this purpose we present a comparative study of the conductance histograms and *I*–*V* characteristics of a selection of solvents commonly used in molecular electronics. We will classify the solvents as “suitable” when the *I*–*V*s of tunnel junctions between tip-like electrodes immersed in the solvent only weakly resemble *I*–*V* curves of single-molecule junctions of functional molecules and therefore enable a clear differentiation between solvent properties and the electronic properties of single molecules. Because of the large variation of possible conductance values and *I*–*V* shapes of single-molecule junctions, we restrict ourselves to the typical conductance range of hydrocarbon-based single-molecule junctions, i.e., 10^−5^*G*_0_ to 10^−3^*G*_0_.

## Single-level model and Simmons model

For many different realizations of tunnel junctions their *I*–*V*s reveal an S-shape. Theoretically this has been worked-out in detail for different shapes of the tunnel barrier by Simmons [30]. For an arbitrary tunnel barrier with average height Φ (measured with respect to the Fermi energy of the non-biased electrode), the current density *J* as a function of applied voltage *V* can be expressed as:

[1]
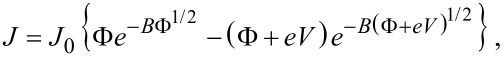


where *J*_0_ and *B* are parameters containing the geometry of the barrier and the mass of the charge carriers and are usually used as fitting parameters. The Simmons model (SM) is widely used for describing the transport through metal/self-assembled monolayer/metal junctions [31] or also applied to single-molecule contacts [32]. However, the quantitative agreement over a larger bias range is often not satisfactory although phenomenological parameters are introduced to improve the fitting [31].

Due to the generality of the model, usually approximate functions are used for low bias, intermediate bias, and high bias. In this context, low, intermediate and high refer to voltage ranges compared to the tunnel barrier height. Here we test the SM up to the intermediate voltage regime |*eV*| < Φ, where for a rectangular barrier the simplified formula

[2]
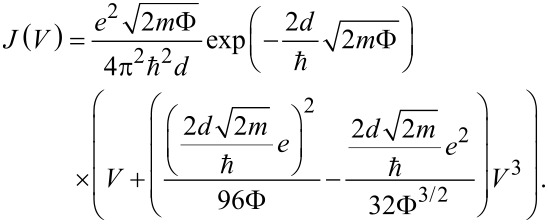


can be applied, with the electron mass m, the reduced Planck’s constant 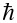, and the thickness of the barrier *d* [30].

To apply the model to atomic-size contacts in which the current, *I*, rather than the current density, *J*, has to be considered, a prefactor A corresponding to the cross section has to be included. Since the size of the cross section is often unknown, it is usually considered as fitting parameter [31].

However, the tunnelling over a barrier is not the only phenomenon that results in S-shaped *I*–*V*s. In fact, the single (resonant)-level transport model (SLM) describes similar shapes in the low- to intermediate-voltage range. The SLM, based on the Landauer picture of electronic transport [2,33], is valid in the case of coherent transport [2,33]. It describes the current as the energy integral over the energy and voltage dependent transmission function of a scatterer:

[3]
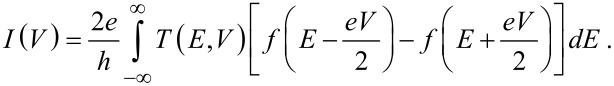


It assumes that the current is carried by an electronic mode that is formed by a single molecular orbital at energy *E*_0_(*V*), coupled to the left and right electrode via the coupling constants Γ_L_ and Γ_R_, respectively. The coupling gives rise to broadening of the level as described by the Breit–Wigner model yielding a resonance with Lorentzian shape for the transmission function *T*(*E*,*V*) [2,12,27,34–37].

[4]
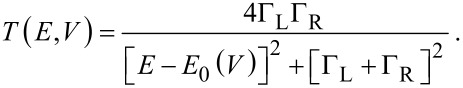


Here, we expect to find symmetric coupling because of the symmetry of the device being formed by two tips of the same metal and of presumably similar shape. In case of symmetric coupling Γ_R_ = Γ_L_ the position of the energy level is independent of the applied voltage. Usually it is assumed that the molecular orbital that is closest to the Fermi energy, i.e., the highest occupied molecular orbital (HOMO) or the lowest unoccupied molecular orbital (LUMO) is the dominating one [2]. When fitting the *I*–*V* curves with this model the energy distance |*E*_0_| of the current-dominating level with respect to the Fermi energy, and the level broadening Γ = (Γ_L_ + Γ_R_)/2 can be inferred from these fits.

When applying larger voltages *eV* > |*E*_0_| or *eV* ≈ Φ, the two models show markedly different behaviour, since in the SLM the current saturates, while according to SM the current diverges exponentially [1–2,30]. In the symmetric SLM the saturation occurs around e*V* = 2|*E*_0_|. However, large voltages are difficult to achieve experimentally, because in general the contacts become unstable, presumably due to heating resulting in current induced or field-induced rearrangements [17,38]. Also such high voltages correspond to high electrostatic fields in which image potential effects must be considered [39]. Therefore for practical reasons the regime of low to intermediate voltages is the most relevant and will be further discussed in this article.

**Figure 1:**
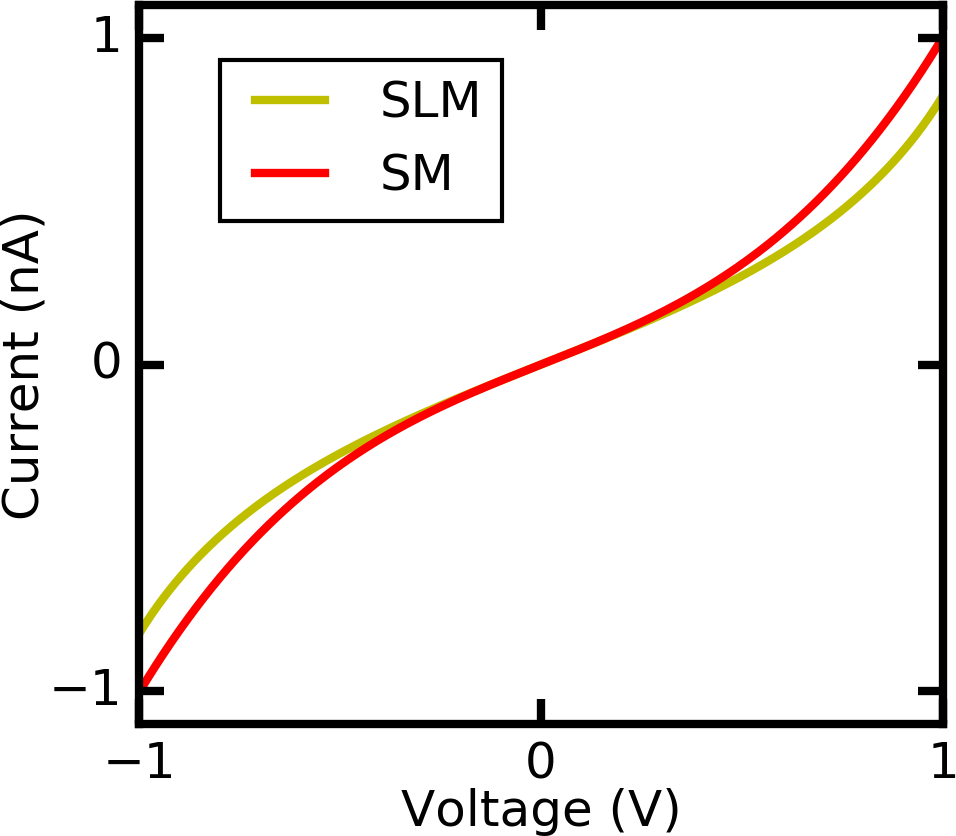
Comparison of Simmons model (SM) and single-level model (SLM) for the parameters Φ = 0.8 eV, *d* = 1.06 nm, *A* = 0.3 nm^2^, and *E*_0_ = 0.8 eV, Γ = 0.001 eV. The linear conductance in both cases corresponds to 6.25·10^−6^*G*_0_.

A particular difficulty arises from the fact that not only the SM (c.f. Equation 2), but also the SLM can be approximated in this regime by a superposition of a linear and a cubic term: *I*(*V*) = *aV* + *bV*^3^ [2,12,30]. However, the ratio between the parameters *a* and *b* is different in both models, when imposing physically motivated limits to the fitting parameters. For instance, |*E*_0_| and Φ have to be in the range of a few hundred millielectronvolts up to a few electronvolts for the solvent molecules under study here. Furthermore, the tunnel distance *d* should be in the range of a few angstroms or nanometres and the cross section *A* in the range of a few square nanometres. In the case of SLM, both *a* and *b* depend on Γ and |*E*_0_|. This bears, in principle, a possibility to distinguish between both models, as exemplified in Figure 1, where we plot two *I*–*V* curves calculated with the SLM and the SM with the level alignment equalling the barrier height. We have chosen here the typical value |*E*_0_| = Φ = 0.8 eV. The other parameters, Γ, *d* and *A* were adapted such that the linear conductance of both models is the same and lies in the order of typical junctions investigated experimentally in this study. As can be clearly seen, for larger voltages |*V*|, the current increases faster in the SM than in the SLM. When fitting the SLM with the SM over the whole range, the SM underestimates the linear conductance, when the nonlinear part (0.5 V < |*V*| < 1 V) is well described.

## Results

**Figure 2:**
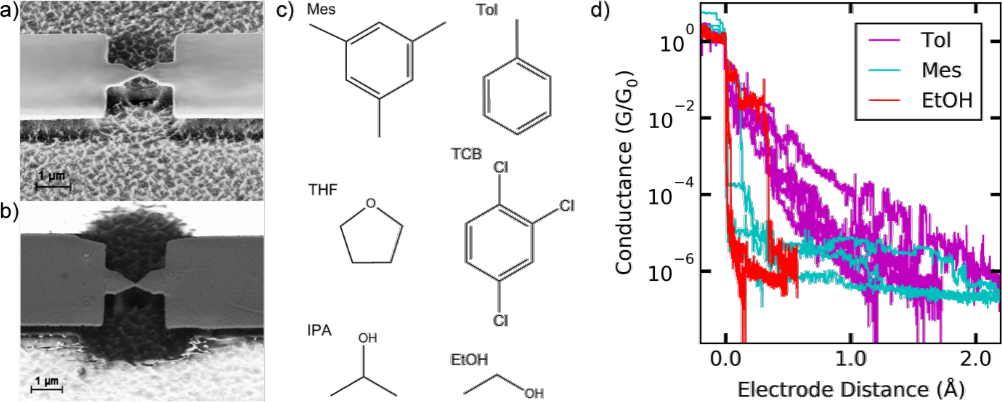
a) Electron micrograph of an Au MCBJ before and b) after measurement in TCB, c) structures of the solvents under study. d) Examples of stretching traces in different solvents.

We use lithographically defined gold electrodes forming freestanding nanobridges on a bendable substrate. Electron micrographs of a sample before and after measurement are shown in Figure 2a and Figure 2b, respectively. The samples are mounted in a custom-designed MCBJ system equipped with a pipette containing the solvent mesitylene (Mes), 1,2,4-trichlorobenzene (TCB), toluene (Tol), ethanol (EtOH), a mixture of 50% tetrahydrofuran and 50% toluene (Tol/THF) or isopropanol (IPA), see Figure 2c. The mixture Tol/THF has been chosen because with pure THF the PDMS gasket of the pipette containing the solution was corroded and became leaky, see Experimental section. However, in pure Tol not all functional molecules were well-dissolved or had a reduced lifetime [11]. After mounting and contacting wires to the samples we measure the conductance as a function of distance under a dc voltage bias of 25 mV to ensure being in the linear conductance regime. The conductance of the pristine junctions amounts to 250–300*G*_0_, where *G*_0_ = 2*e*^2^/*h* denotes the conductance quantum. Upon stretching the metallic bridge, the conductance decreases stepwise and the last single-atom Au–Au contact breaks at a conductance *G* ≈ 1*G*_0_. Upon further stretching the conductance decreases smoothly with roughly exponential distance dependence, superimposed by fluctuations and irregular steps, typical for measurements using the MCBJ technique in solution. In Figure 2d we show examples of stretching curves for Mes, Tol and EtOH. Obviously, for Mes the conductance decays on a much shorter distance than for Tol. This hints at a solvent-dependent variation of the work function of Au [9–10]. The fluctuations of the conductance show a larger amplitude for Tol than for EtOH and Mes despite the fact that the identical electrical set-up and bias voltage have been used. This indicates that the temporal fluctuations of the contact geometry are more pronounced for Tol and EtOH than for Mes. Furthermore, in the individual breaking curves of EtOH plateaus are observed more frequently than for the other solvents. Plateaus are signatures of the formation of molecular contacts. Here they occur in particular at small opening distance, in agreement with the small size of the solvent molecules. The lowest conductance value that we are able to detect is around 10^−9^*G*_0_ in agreement with reports from other groups [12,40]. In some solvents we observe levelling-off of the minimum conductance value around 10^−6^*G*_0_, caused by electrochemical effects occurring in the junction, as we will detail below.

**Figure 3:**
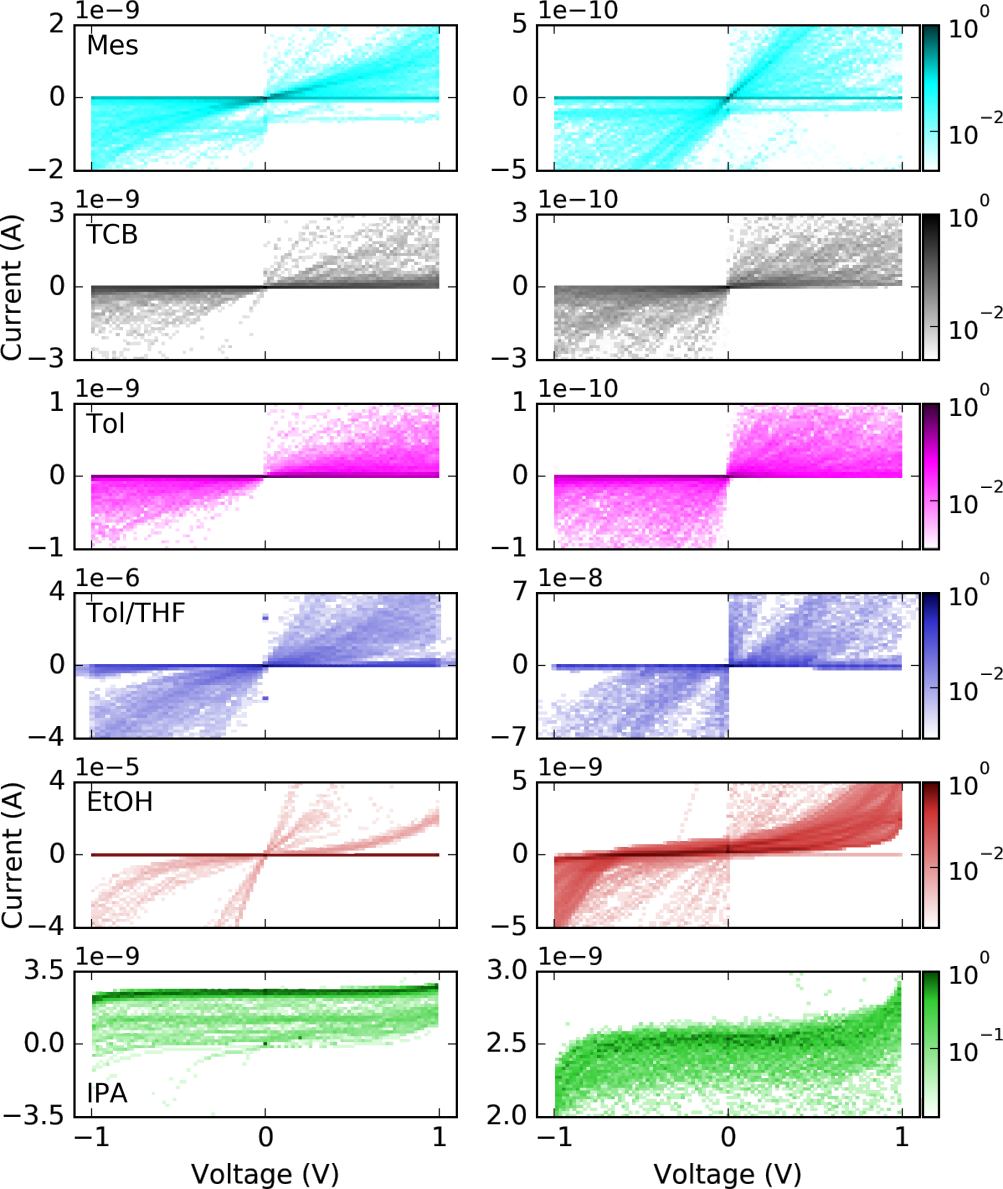
Density plots of current–voltage characteristics of all investigated solvents. The right column gives a close up for the small current region. The colour code is normalized. The total numbers of curves are: Mes 107, TCB 70, Tol 173, Tol/THF 180, EtOH 372, IPA 55. Note different scales for current axes.

When the minimum conductance is achieved, the bending of the substrate is released and the stretching procedure is reversed, relaxing the junction back until Au–Au contacts with a conductance of roughly 50*G*_0_ are achieved. For recording the *I*–*Vs* the stretching process is stopped. This can be done at any position of the stretching or relaxing trace and in a wide conductance range. We then first set the voltage to zero, subsequently ramp it up to +1 V, decrease it to −1 V and finally sweep it back to zero while the current is monitored. We record up to several hundred *I*–*V*s for each solvent. Depending on the solvent and the conductance, in few or many cases, the conductance at the end of the sweep is different from the initial one, indicating that the geometry of the junctions was modified during the sweep. We evaluate those *I*–*V*s in which the conductance at start and at end differ by less than 20% from each other. We also restrict the analysis to symmetric *I*–*V*s, defined such that the current amplitude at positive and negative bias also agree within 20% with each other, because asymmetric *I*–*V*s hint at asymmetric contacts that are not expected to occur for transport through solvent with identical material and shape of the electrodes. Furthermore, for describing asymmetric *I–V*s additional fitting parameters, i.e., differing tunnel barrier heights, Φ_L_ and Φ_R_, in the SM or coupling constants Γ_L_ and Γ_R_ in the SLM have to be considered. This makes a meaningful determination of these parameters impossible, given the experimental scatter at room temperature. This restriction further reduces the number of usable *I*–*V*s to the values indicated in Table 1. In Figure 3 we plot the *I*–*V*s as density plots, in which the number of data points is colour-coded, darker colours indicate a higher number of counts.

**Table 1:** Statistics regarding the molecular properties and the fitting of the *I*–*V* characteristics with the SLM and the SM. The numbers indicate the total number of *I*–*V*s recorded for the individual solvents (total), the number of *I*–*V*s showing pronounced hysteresis (open), the number of *I*–*V*s that can be fitted with the SLM, the SM, both or none of the models as well as the number of S-shaped but asymmetric *I*–*V*s (asym). The number of *I*–*V*s with jumps or kinks or other irregularities that, therefore, did not fulfil the stability criteria, are summarized in the last column (other).

solvent	total	open	SLM	SM	both	none	asym	other

Mes	107	9	19	20	14	31	39	3
TCB	70	4	7	5	4	18	28	12
Tol	576	4	11	6	5	67	85	408
Tol/THF	180	0	1	1	0	83	23	72
EtOH	430	380	3	4	3	17	22	7
IPA	55	54	0	0	0	1	0	0

Figure 3 reveals that depending on the solvent the stable S-shaped *I*–*V*s were recorded in different current regimes. While for IPA, Mes, TCB, and Tol these are dominantly found in the low current regime *I* < 1 µA, EtOH and Tol/THF reveal stable *I*–*V*s with currents up to 100 µA. A phenomenon most prominent for EtOH and IPA, and with much lower probability and amplitude also for Mes, TCB and Tol is the appearance of “open loops” and current offsets, i.e., *I*(*V* = 0) ≠ 0, with higher currents during increasing than during decreasing the voltage. For Mes, Tol and TCB these open loops and offsets are only seen in the low-current regime (below 10^−10^ A). For EtOH and IPA the offset current is higher: 10^−9^ to 10^−8^ A. The open-loop effects develop non-uniformly with time when continuously stretching a sample and may disappear abruptly when having closed the contact again. For all solvents except for IPA it was also possible to record *I*–*V*s without current offset at zero voltage. The open-loop curves were discarded from further analysis, thus reducing the number of *I*–*V* curves. We will discuss possible origins of this effect further below.

We now turn our analysis to the fitting of the experimental *I*–*V*s with the two models. For tunnelling through a solvent environment one would not expect to have transport through a unique molecular orbital with well-defined coupling constant, unless a solvent molecule anchors to the electrodes and forms a single-molecule contact. We therefore try to fit all stable *I*–*V*s of all solvents to both models to test how well they can be reproduced by the models and if they can therefore be mistaken as junctions formed by functional molecules.

**Figure 4:**
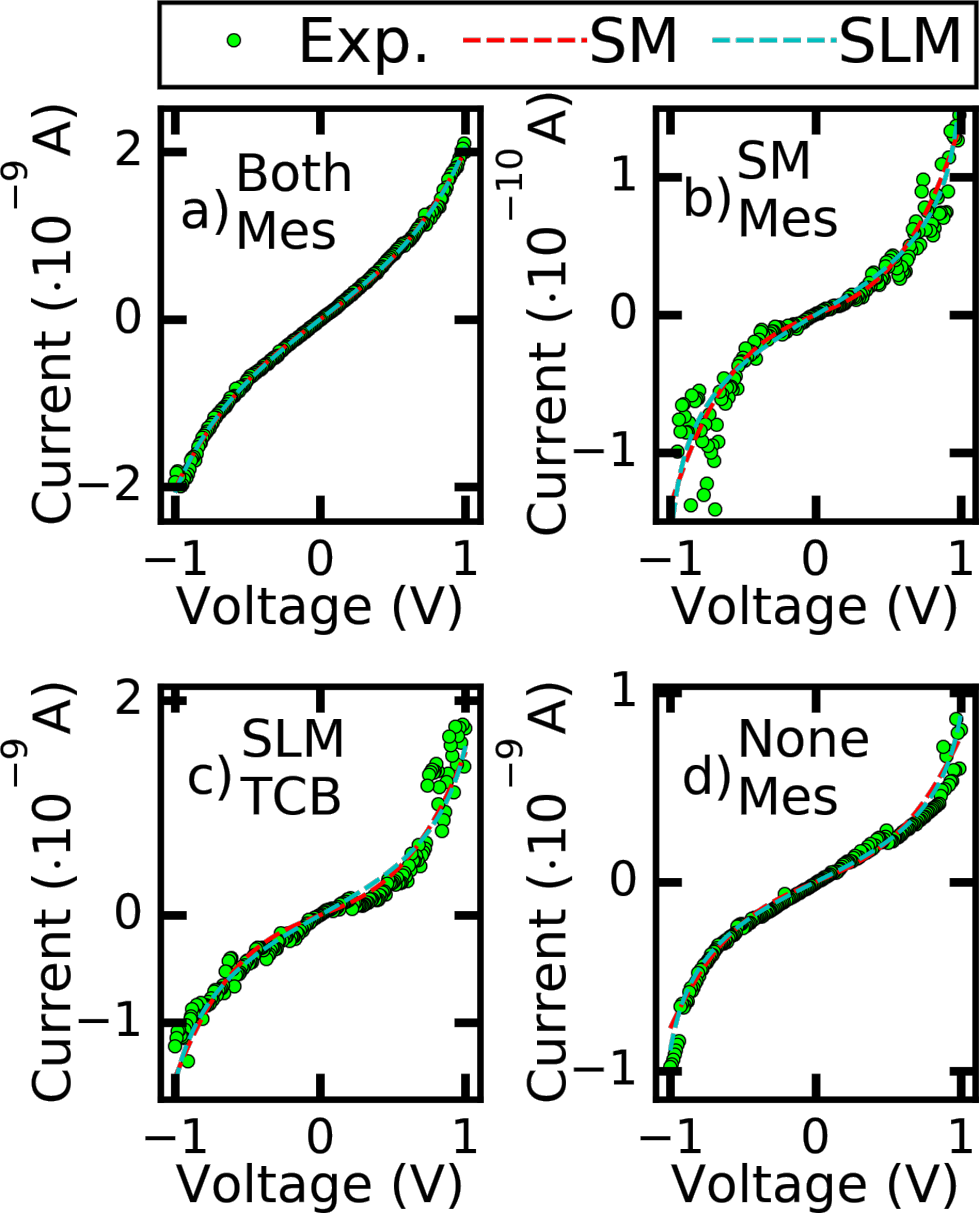
Examples of *I*–*V*s and their best-fittings to the SLM and the SM. a) Fitting with both models, |*E*_0_| = 0.867 ± 0.008 eV, Γ = 1.81 ± 0.02 meV, Φ = 1.001 eV ± 0.018 eV, *d* = 0.88 ± 0.070 nm, *G*_exp_ = (1.736 ± 0.007)·10^−5^*G*_0_, *G*_SLM_ = 1.758·10^−5^*G*_0_, *G*_SM_ = 1.673·10^−5^*G*_0_; b) Fitting solely with the SM. |*E*_0_| = 0.707 ± 0.005 eV, Γ = 0.315 ± 0.071 meV, Φ = 0.694 ± 0.070 eV, *d* = 1.383 ± 0.072 nm, *G*_exp_ = (6.08 ± 0.29)·10^−7^*G*_0_, *G*_SLM_ = 8.06·10^−7^*G*_0_, *G*_SM_ = 5.60·10^−7^*G*_0_, c) Fitting solely with the SLM. |*E*_0_| = 0.727 ± 0.014 eV, Γ = 1.13 ± 0.040 meV, Φ = 0.564 ± 0.046 eV, *d* = 1.21 nm ± 0.05 nm, *G*_exp_ = (9.91 ± 0.58)·10^−7^*G*_0_, *G*_SLM_ = 9.85·10^−7^*G*_0_, *G*_SM_ = 6.78·10^−6^*G*_0_, d) Not fitting with either model. *|E*_0_| = 0.709 ± 0.005 eV, Γ = 0.78 ± 0.01 meV, Φ = 0.715 ± 0.038 eV, *d* = 1.15 ± 0.03 nm, *G*_exp_ = (5.98 ± 0.06)·10^−6^*G*_0_, *G*_SLM_= 4.42·10^−6^*G*_0_, *G*_SM_ = 4.15·10^−6^ *G*_0_. The errors indicate the numerical error deduced from the least squares fitting.

In Figure 4 we exemplify *I*–*V*s and their fittings to the SLM and the SM model. The examples we show are from different solvents to give an overview over the typical behaviour. The examples show the four possible cases: a) Fitting to both models, b) fitting solely with the SM, c) fitting solely with the SLM, d) not fitting to either model, according to the criteria given below. Figure 4 reveals that in general the scatter increases with bias, revealing that it arises from the intrinsic motion of the junctions and not from the finite electrical measurement resolution. However, this scatter limits the precision with which the models can be fitted to the data. We apply a standard least squares fitting algorithm. As a measure for the fit quality we use χ^2^ of the fit as well as the resulting error bars for the fit parameters deduced from the number of combinations of fit parameters fulfilling the fitting criteria. Fits are qualified as fitting if, in addition, the following criteria are fulfilled: the slope of the *I*–*V* around zero-bias should be reproduced well by the model (within 20% (25%) error for the SLM (SM)). We chose the stronger criterion when fitting with the SLM, because the wider one would have yielded basically the same data set. However, when fitting with the SM, only very few curves fulfil the 20% criterion (less than half of the ones that lie within 25%). When further relaxing the criterion the errors of the best-fit results increase enormously. Examples of fitting curves that failed because they do not meet the linear-slope criterion are the SLM in Figure 4b and Figure 4d and the SM in Figure 4c and Figure 4d. Second, the functional shape of the *I*–*V* should be reproduced well by the fitting function. This is not straightforward to implement numerically in view of the increasing fluctuations at higher currents. We therefore manually discarded those curves in which the data points deviate systematically from the fit curve in a certain voltage range. An example is given in Figure 4d, where the SM fails to describe the data in the voltage ranges −1V < *V* < −0.8 V and 0.6V < *V* ≤ 0.8 V. Altogether these criteria are less strict than those which are imposed on the fit quality for functional molecules, where the thresholds are 10–15% for the linear conductance and the conductance at the outer ends of the voltage range [11–12,16–17,27,34–36,41]. Here we chose a wider range to ensure that all solvent contacts that could be mistaken for molecular contacts are detected.

**Figure 5:**
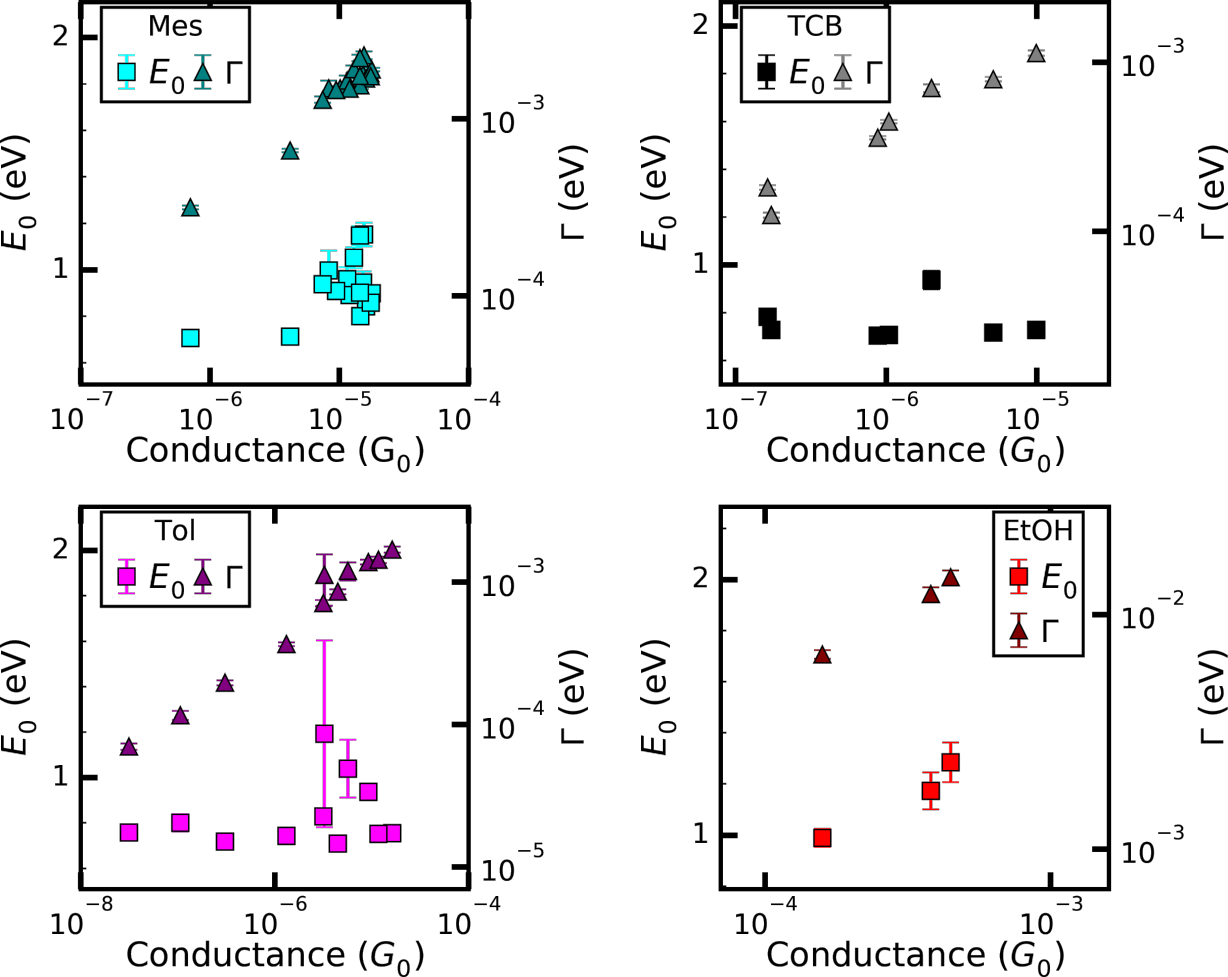
Fitting results using the SLM (Equation 3 and Equation 4). The error bars denote the numerical error of the least-squares fitting of the individual curves.

**Figure 6:**
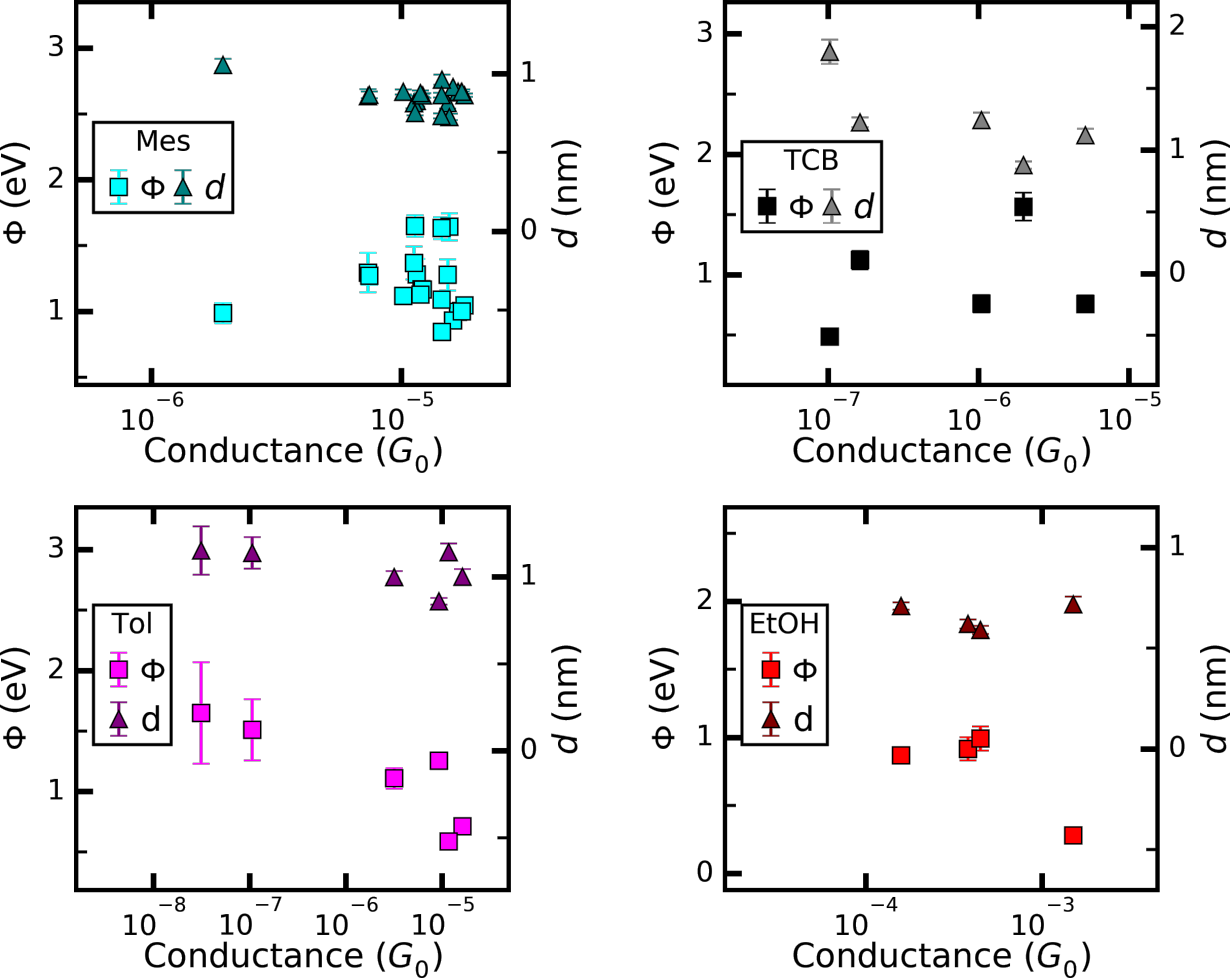
Results of fitting with SM, Equation 2. The error bars denote the numerical error of the least-squares fitting of the individual curves.

The values extracted from fitting to the SLM for all solvents are plotted in Figure 5 and those to the SM in Figure 6 as a function of the linear conductance (determined from the slope of the *I*–*V* for |*V*| < 0.1 V) and their average values are summarized in Table 2. No graph is shown for those solvents where only up to two curves could be fitted.

**Table 2:** Averaged fitting results with SLM and SM, and dielectric properties of pure solvents [42–43]. Here the error ranges denote the statistical error from the averaging over all fit results. The values marked with an asterisk are given for THF. The properties of the respective solvents are: µ the dipole moment, ε_r_ the low frequency dielectric constant, and σ the DC conductivity. Data taken form the Reaxys® data base and references therein.

name	Γ (meV)	*|E*_0_| (eV)	Φ (eV)	*d* (nm)	µ (D)	ε_r_ (F·m^−1^)	σ (S·cm^−1^)

Mes	1.73 ± 0.26	0.93 ± 0.10	1.20 ± 0.23	0.86 ± 0.07	0.047	2.3	2·10^−17^
TCB	0.54 ± 0.33	0.76 + 0.08	0.94 ± 0.37	1.25 ± 0.30	1.26	2.24	10^−11^
Tol	0.83 ± 0.55	0.84 ± 0.15	1.14 ± 0.39	1.05 ± 0.11	0.375	2.4	8·10^−16^
Tol/THF	14.5 ± 0.4	0.70 ± 0.11	0.080 ± 0.002	1.27 ± 0.25	1.75*	7.5*	10^−6^*
EtOH	11.1 ± 3.2	1.15 ± 0.12	0.76 ± 0.28	0.66 ± 0.06	1.69	24.6	1.4·10^−9^
IPA	—	—	—	—	1.66	18	6·10^−8^

Figure 5 reveals that Mes, TCB, Tol, and EtOH show no pronounced dependence of the level alignment |*E*_0_| on the conductance, however, the variation of the best-fit results increases with conductance. This means that the onset of the non-linear contribution in the *I*–*V*s is independent of the transmission and justifies the recording of opening traces under constant voltage bias, as usually done in the experiments. For EtOH, the scatter of |*E*_0_| is the largest, suggesting that the behaviour of junctions in EtOH is more complex than in the other investigated solvents. For all solvents the increase of the conductance is governed by an increase of the coupling strength Γ that rises from roughly 0.1 to 1 meV, smaller than typical coupling constants for single-molecule junctions of functional molecules [11,27,34,41]. The average values for Γ of Tol/THF and EtOH (see Table 2) are much higher than those of the other solvents, because for these two solvents the *I*–*V*s that were successfully described by the SLM were situated in a higher conductance regime.

We now turn to the fitting results with the SM. To limit the number of fitting parameters the cross-section of the junctions, described by the prefactor *A* in the SM was fixed to *A* = 0.3 nm^2^, corresponding to tip radii of 1–3 nm typical for MCBJ contacts. This further reduces the number of *I*–*V* curves that can be described by the SM. As a result only four *I*–*Vs* for EtOH and only one for Tol/THF could be fitted. For Tol/THF the value obtained for the tunnel barrier height Φ lies in the range around 0.1 eV. Since the validity of the approximation in Equation 2 is limited to e*V* < Φ, this result is not meaningful. Thus, we find that the junctions investigated for Tol/THF cannot be described by the SM in the used approximation. They might correspond to junctions formed by blunt tips. For the other four solvents, Mes, TCB, Tol, and EtOH, Φ is in the range of 0.5 to 1.5 eV, with one exception for a high-conductance curve (*G* > 10^−3^*G*_0_) of EtOH, where also a rather small Φ is observed.

Overall, the Φ values are roughly 20% higher than the |*E*_0_| values, but show somewhat larger scatter and more variation from solvent to solvent. When comparing the fit parameters obtained on those curves that can be described with both models, |*E*_0_| and Φ typically agree within 10% with each other as expected by the physical meaning of these parameters. The tunnel distances are in the range of 0.8 to 1.3 nm and slightly decrease with increasing conductance as expected, however, the scatter is much higher than the overall decrease. Note that the error bars in Figure 5 and Figure 6 account for the numerical error of the fit procedures only. The variations of the fit results, in particular those of |*E*_0_| and Φ are much larger than the error bars, indicating that these parameters are determined by microscopic properties of the junctions, i.e., by the atomic configurations.

## Discussion

As mentioned above, we consider a solvent as “suitable”, if its transport properties in MCBJ geometry cannot be mistaken for a single-molecule junction formed by a “functional” molecule dissolved in the respective solvent. In addition the solvent should not cause artefacts in the *I*–*V*s or stretching curves and, obviously, it must be able to dissolve the functional molecules without deteriorating it and without precipitations. To be specific we apply the following criteria: 1. the abundance of open-loop effects; 2. the percentage of *I*–*V*s being well described by the SLM; 3. the similarity of best-fit parameters with the SLM or SM with those of typical functional molecules; 4. the probability of step-formation in the stretching curves. For a given solvent, all these values should be small to qualify it as a suitable solvent.

We now discuss the properties of the studied solvents according to these criteria. THF, IPA and EtOH have considerably higher conductivity than the other three solvents, see Table 2. This is in agreement with the observation that for these solvents junctions in the higher conductance range were more abundant. However, the *I*–*V*s of these junctions were mostly not well-fitted with the SLM or the SM. If one estimates the conductance using the bulk formula: *G* = σ·*A*/*d* and using the values *A* = 100 × 100 nm^2^ for the cross-section (corresponding to the cross section of the electrodes before break) and *d* = 1 nm for the length of the conductor (estimated from the fitting results with the SM), the resulting values for the conductance are of the order of 10^−5^*G*_0_ (for THF), 10^−6^*G*_0_ (for IPA) and 10^−8^*G*_0_ (for EtOH), i.e., smaller than the conductance of the individual junctions analysed in detail here. This estimation suggests that the high-conductance junctions formed in IPA and Tol/THF might reveal contributions of direct conductance through the solvent, while pure EtOH may still be considered as tunnel barrier.

EtOH and IPA show the open-loop effect most prominently, with the effect that the majority of the *I*–*V*s could not be fitted to the models. No *I*–*V* curve recorded for IPA could be fitted by either the SLM or the SM. However, the total number of curves is low, since the MCBJ samples showed a high failure rate when used in IPA. They did not close to a conductance above 1*G*_0_ again after a small number of stretching and relaxing cycles. Those curves of EtOH that show no open-loop effect can be well described with the SLM but less well with the SM in a conductance range of 10^−5^*G*_0_ to 10^−2^*G*_0_. Although the ratio of S-shaped *I*–*V*s is small for both solvents, artefacts such as open-loops may occur also when functional molecules are contained. Thus, when EtOH is used as solvent for single-molecule experiments, it has to be used with caution.

*I*–*V*s recorded in TCB show almost no open-loop effects and can, to a small ratio, be described by the SM or by the SLM. The S-shaped, fitting *I*–*V*s occur in a conductance range of 10^−7^*G*_0_ to 10^−5^*G*_0_, partially overlapping with the region of interest of functional single-molecule junctions. Below 10^−5^*G*_0_ some of the |*E*_0_| values are close to those of functional molecules, hampering a clear distinction between solvent and molecular junctions, although more than half of those S-shaped *I*–*V*s in the range above 10^−6^*G*_0_ cannot be fitted. We observe a tendency of destabilization of the contacts in the electrical field, revealing itself by enhanced scatter at high voltages, despite relatively low currents. Thus, TCB seems to be a suitable solvent for single-molecule transport studies for molecular junctions in the conductance range above 10^−4^*G*_0_ only.

The percentage of open-loop *I*–*V*s of Tol is comparable with the one of TCB. Most curves cannot be fitted, neither with the SLM nor with the SM, although S-shaped *I*–*V*s may appear in a wide conductance range of 10^−9^*G*_0_ to 10^−1^*G*_0_. The ratio of S-shaped curves is reduced in the intermediate-conductance range around 10^−3^*G*_0_. In the low-conductance range *G* < 10^−5^*G*_0_, a considerable part of the *I*–*V*s can be fitted with both the SLM and the SM, while none can be fitted in the high-conductance range *G* > 10^−3^*G*_0_. Therefore Tol seems to be a suitable solvent for single-molecule studies in this range.

Compared to Tol and TCB, Mes reveals a slightly higher ratio of open-loop curves, however, with small size of the offset current and restricted to the low-conductance range, *G* < 10^−7^*G*_0_. In addition, the number of unstable curves revealing kinks or high scatter is very low, reflecting the low chemical activity of Mes. Therefore, a relatively high number of the remaining S-shaped symmetric curves, roughly 20%, can be described well by the SM and by the SLM simultaneously. In our study most of the S-shaped curves were observed around 10^−5^*G*_0_, although some *I*–*V*s were measured in a much wider range of 10^−8^*G*_0_ to 10^−3^*G*_0_. However, for lower or higher conductance these *I*–*V*s had different shapes. In addition the sampling was not homogeneously spread over the conductance range, because we have concentrated on the range around 10^−5^*G*_0_ in view of a particular functional molecule that was studied [16]. The average |*E*_0_| and Φ values are higher than those of typical functional molecules. In combination with its low toxicity it therefore appears as a suitable choice.

Tol/THF reveals many S-shaped *I*–*V*s in the conductance range of single-molecule contacts, out of which only one can be fitted to either model. These curves correspond to a relatively high conductance of *G* > 10^−4^*G*_0_. A considerable part of the *I*–*V*s is asymmetric, suggesting the formation of junctions in which solvent molecules are bound more strongly to one of the electrodes than to the other. Therefore, the interpretation of single-molecule transport data in Tol/THF needs particular caution, but with a critical analysis of the *I*–*V*s also this mixture of solvents appears as a suitable choice.

To further elucidate the different behaviour of the solvents we first discuss possible origins of open loops and offsets. One apparent possibility would be capacitive effects. We estimated the geometrical capacitance of the MCBJ electrodes with respect to the ground plate to roughly 20 pF, thus much too low to explain the pronounced loop opening for the scanning speeds (40 seconds per *I*–*V* sweep) used here. The effective capacitance of the junction might be enhanced by the capacitance of the nano-contact, which is embedded in the solvent. Since the sample design and the measurement set-up and electronics were the same for all solvents, the sample specific parameters, i.e., the dielectric constant ε_r_ and the bulk conductivity σ of the solvent, have to be considered, see Table 2. Furthermore, capacitive effects cannot explain the finite offset (*I*(*V* = 0)) that is bigger than the opening of the loop (current difference between up- and down-sweep) observed in EtOH and IPA. Thus, although EtOH and IPA have a high ε_r_ around 20 followed by THF with ε_r_ ≈ 7.5 while Mes has ε_r_ = 2.23, this cannot be the only explanation. In addition, the offset current did not depend on the sweeping speed, as opposed to capacitive loop-opening. Another option would be dipolar effects. As Table 2 reveals IPA, THF, EtOH and TCB have rather elevated dipole moments above 1.2 D, out of which only IPA and EtOH show the pronounced open-loop effect. Therefore, there is also no strict correlation between the dipole moment and the offset current, although dipolar effects have been reported in recent experimental and theoretical work to be able to tune the conductance markedly [13,21]. While we cannot exclude dipolar effects to be active, we conclude that electrochemical processes in the junction, as known from cyclic voltammetry [23], e.g., oxidation and reduction of the solvent, must play a role as well [44–45]. This interpretation is supported by the fact that the offsets do not occur in all cases for a given solvent. Electrochemical processes are induced by the local electric field, the size of which depends on the shape of the electrode tips. We suggest that protic solvents such as EtOH and IPA might as well get deprotonated at the tips. As a result the tips become contaminated faster by hydrogen and other constituents of the molecules. We observe a tendency of a reduced lifetime of electrodes in protic solvents as compared to the other solvents, being most prominent for IPA. Here, the lifetime is measured by the number of *I*–*V* measurements that can be recorded before the sample fails. While with samples immersed into Tol/THF or Tol several hundred *I*–*V*s can be taken, samples immersed into IPA or EtOH rarely sustain more than 20 *I*–*V*s.

**Figure 7:**
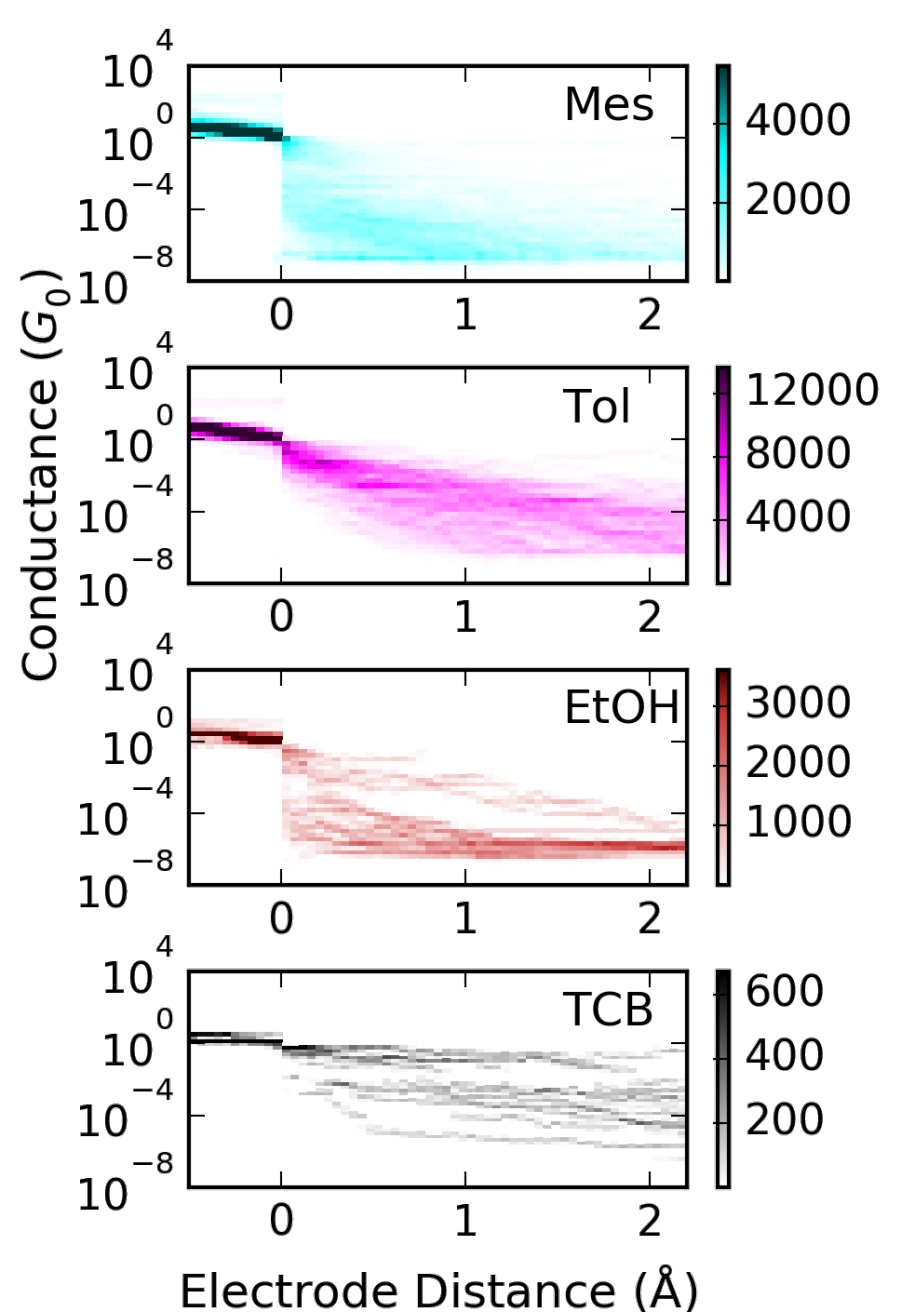
Two-dimensional histograms (density plots) of Au MCBJs in Mes, Tol, EtOH, and TCB calculated from stretching traces. The individual traces have been overlaid at a conductance of 0.5*G*_0_. The numbers of curves are: Mes 649, Tol 186, EtOH, 26, TCB 19.

Finally we address the shape of the breaking traces and histograms for Mes, EtOH, Tol and TCB, see Figure 7. A fast decrease is observed for EtOH and Mes. For Mes in the high-conductance range 10^−2^*G*_0_ < *G* occasionally a step-like behaviour occurs, see also Figure 2, indicating the trapping of molecules in the junctions. From the literature it is known that in Mes contacts with a conductance of *G* ≈ 0.1*G*_0_ and with G ≈ 0.03*G*_0_ may form [18–19]. This effect appears more frequently upon ageing of the samples and is therefore attributed to the increasing water content with time [45]. Contamination with water would also explain the appearance of the hysteresis discussed above. For Tol and TCB occasionally very long breaking traces are observed that indicate the formation of molecular junctions or even stacking of solvent molecules [16]. The observation of fast decrease of conductance corresponds to a high tunnel barrier Φ or a high |*E*_0_|, respectively. For Mes this finding is in agreement with the fitting results to the SM. For EtOH the SM did not result in a meaningful description of the *I*–*V*s, however, the few curves that were possible to fit with the SLM also reveal a high |*E*_0_|.

Summarizing this study, Mes, Tol/THF and Tol seem to be the most suitable solvents for the study of single-molecule junctions of organic molecules. Of course other criteria, as for instance the solubility of the molecules, the probability with which a stable molecular contact can be formed, the long-time stability of the contacts, or the lifetime of Au MCBJs when exposed to the solvent may also be considered. While the lifetime aspect is shortly addressed in our work, a comprehensive study of all these aspects is beyond the scope of this article.

## Conclusion

We have investigated charge transport through tunnel contacts formed by the mechanically controllable break-junction method at room temperature in different solvents. The conductance has been recorded while stretching and relaxing the molecular junctions repeatedly revealing indications for the formation of junctions incorporating solvent molecules or fragments of the molecules. By analysing the *I*–*V* curves with the help of the single-level model and the Simmons model for tunnelling through a barrier, we discuss their suitability as solvents in single-molecule studies of organic molecules. We consider a solvent as suitable, if its transport behaviour can be distinguished from the one of junctions formed by functional molecules dissolved in the solvent and bound to the electrodes. Our analysis reveals that mesitylene (Mes), toluene (Tol) and a mixture of toluene and tetrahydrofurane (Tol/THF) seem to be the best choice. Mes has advantages compared to Tol in view of lower toxicity. Although in our experiments we did not find unambiguous evidence for polar effects, also in this respect the very low dipole moment of Mes appears favourable.

## Experimental

### Purification of solvents

All solvents were acquired from commercial suppliers (Sigma Aldrich and Merck KGaA) and were of analytical grade (>99.5%). Solvents were further purified and dried by standard laboratory methods prior to use [46] and stored under nitrogen inert gas atmosphere if necessary. EtOH of content greater than 99.5% was dried over activated magnesium turnings and distilled and stored under inert gas atmosphere. Tol, Mes and THF were distilled from sodium/potassium alloy under inert gas atmosphere. TCB was dried over P_4_O_10_ and distilled under nitrogen inert gas atmosphere.

### Device fabrication

As described in [11] the spin-coating of polyimide (2 μm in thickness) is performed on a softly polished bronze wafer (200 μm in thickness), and then the wafer is annealed for 6 h at 430 °C in vacuum (10^−5^ mbar). The polyimide layer serves as an electrical insulator and a sacrificial layer in the subsequent etching process. Prior to performing the electron beam lithography performed in a FEI scanning electron microscope XL30 equipped with a pattern generator (Raith Elphy Quantum), a double layer of electron-beam resists (MMA-MAA/PMMA) is deposited by spin-coating on the wafer to a thickness of about 600 nm (MMA-MAA) and about 100 nm (PMMA) and baked on a hot plate (130 °C for 5 min). After electron beam writing and developing in MIBK:IPA (1:3) and rinsing in IPA, the patterned samples are mounted in an electron-beam evaporator of ultra-high vacuum (10^−9^ mbar) and gold of about 80 nm thickness is deposited at a rate of 1 Å/s. After lift-off, the polyimide layer is partially etched away (thickness reduction ca. 700 nm) by employing O_2_ plasma in the vacuum chamber of a reactive ion etcher in order to form a free-standing bridge [26,28].

### Break junction setup for measurements of molecular contacts in solution

**Figure 8:**
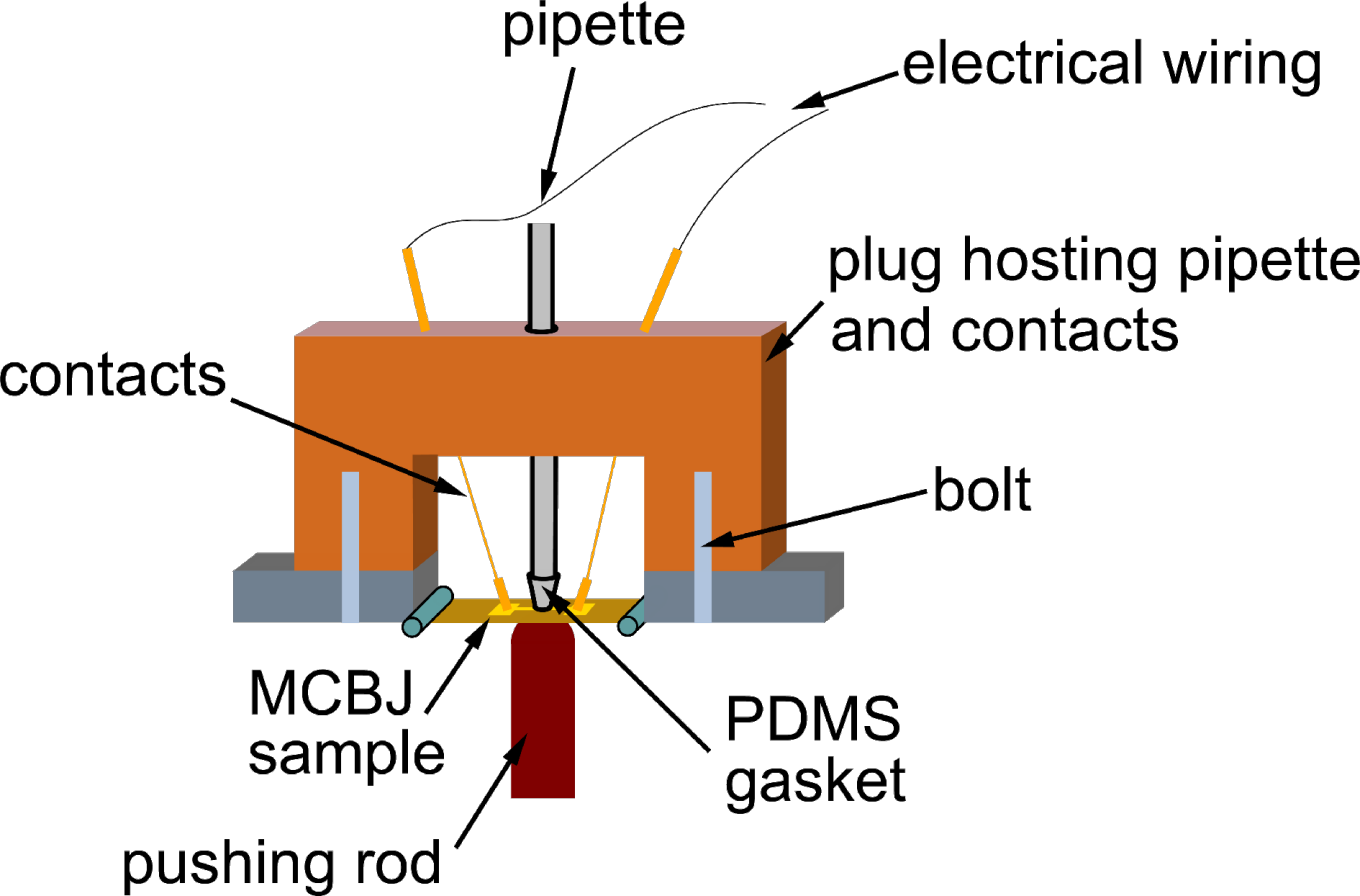
Break junction set-up for use in liquid environment. A PDMS-sealed glass pipette, in which the molecular solution circulates, is pressed onto the central part of MCBJ chip with the help of a plug screwed to the sample holder. The electrical contacts are realized in this case via spring-borne contacts outside the gasket. In some experiments the contact between the contact tips and the sample was mediated by small indium platelets.

As described in [11], the samples are mounted onto the three-point bending mechanism shown in Figure 8. The electrodes are contacted by lowering the spring-borne contacts onto the pads. The droplet, about 1–2 mL, of the respective solvent is injected into the PDMS sealed pipette and carefully lowered onto the electrode device [10–11]. The set-up is installed in a closed metal case for shielding high frequency noise.

The breaking mechanics is controlled by a DC motor with position sensor (Faulhaber, model 22/2, reduction ratio 1:1734) driving a rotary axis, see Figure 8. The rotation of the axis is transformed into a lateral motion of a pushing rod using a differential screw. The motor position is then translated into an axial motion of the pushing rod. The interelectrode distance change (Δ*u*) is estimated from the displacement of the pushing rod (Δ*z*) via an attenuation factor (*r*): ∆*u* = *r*·∆*z*, where *r* = ξ·6*tu*/*L*^2^. Here, *t* ≈ 0.25 mm is the thickness of the substrate, *u* ≈ 2 μm is the length of the free-standing bridge, *L* = 16 mm is the distance of the counter supports, and ξ is a correction factor which has a value varying from 2 to 4 depending on details of the sample [47]. *r* can be determined experimentally from conductance–distance curves in vacuum, when the work function of the electrode is known. By using the value Φ_0_ = 5.1 eV for Au, we estimate ξ = 4 for our samples. However, since the distance *u* varies from sample to sample as well, the distance values bear an uncertainty of roughly 50%.

### Electrical measurement

All electrical measurements are performed at room temperature in liquid environment. The conductance measurements of stretching and relaxing were performed by using a custom-built logarithmic amplifier [48–49].The *I*–*V* curves have been recorded by a sub-femtoamp source-meter (Keithley 6430) operating with an automatic variable gain pre-amplifier. The voltage has been swept usually at a rate of 100 mV/s. Smaller rates have been tested when open-loop effects occurred. Every grounds of the system were carefully designed to avoid ground-loops and electrical noise. All data were collected by a LabVIEW software through GPIB cables.
